# *Bacillus velezensis* Ag129 and Ag132: two novel probiotics enhancing drought tolerance and agronomic performance in maize and soybean

**DOI:** 10.3389/fpls.2026.1711687

**Published:** 2026-02-13

**Authors:** Antoni Wallace Marcos, Juarez Pires Tomaz, Alison Fernando Nogueira, Mirela Mosela, Daniel Soares Alves, José dos Santos Neto, Lycio Shinji Watanabe, Leandro Afonso, Marcos Ventura Faria, Liliane Scislowski, Daniel Fernando Viana Fagundes, Henry Boguschi Cava, Pablo Diego Silva Cabral, Roger Wisniewski da Conceição, Rafael de Assis, Sérgio Vicente de Azevedo, Liliam Silvia Candido, Leandro Simões Azeredo Gonçalves

**Affiliations:** 1 Agronomy Department, Universidade Estadual de Londrina (UEL), Londrina, Paraná, Brazil; 2 Plant Breeding and Propagation Area, Instituto Rural do Paraná (IDR-Paraná), Londrina, Paraná, Brazil; 3 Microbiology Department, Universidade Estadual de Londrina (UEL), Londrina, Paraná, Brazil; 4 Agronomy Department, Centro Universitário Filadélfia (UNIFIL), Londrina, Paraná, Brazil; 5 Chemical Departament, Universidade Estadual de Londrina (UEL), Londrina, Paraná, Brazil; 6 Agronomy Department, Universidade Estadual do Centro Oeste (UNICENTRO), Guarapuava, Paraná, Brazil; 7 Agronomy Department, Instituto Federal Goiano (IFG), Rio Verde, Goiás, Brazil; 8 Biology Department, Instituto Federal de São Paulo (IFSP), Barretos, São Paulo, Brazil; 9 Biology Department, Universidade Federal de Grande Dourados (UFGD), Dourados, Mato Grosso do Sul, Brazil

**Keywords:** abiotic stress, *Arabidopsis thaliana*, *Glycine max* L., inoculants, *Phaseolus vulgaris* L. plant growth-promoting rhizobacteria (PGPR), water scarcity, *Zea mays* L.

## Abstract

Water deficit is one of the main challenges to yield stability in tropical agricultural systems. This study aimed to identify bacterial strains capable of promoting plant growth and mitigating drought effects across different crop species. Initially, eight *Bacillus* strains were evaluated under water-deficit conditions in both growth chamber (*Arabidopsis thaliana*) and greenhouse experiments (common bean, soybean, and maize). Based on agronomic performance, two strains (Ag129 and Ag132) were selected for further validation under field conditions in different edaphoclimatic conditions. In maize, mean yield increases of 10.01% and 8.79% were observed for Ag129 and Ag132, respectively. In soybean, the average gains were 10.82% and 15.20% relative to the uninoculated control, with broad production stability across environments. Genome sequencing identified both strains as *Bacillus velezensis* (4,046,556 and 4,039,722 bp; GC 46.14% and 46.22%). We annotated 3,005 and 3,169 coding sequences, of which 2,861 and 3,011 were assigned to 22 COG categories. KEGG mapping allocated 2,259 and 2,369 genes to 252 and 265 pathways, respectively. Functionally, both genomes harbor a broad repertoire of plant growth-promoting traits relevant to drought resilience, including responses to abiotic stress, phytohormone biosynthetic potential (indole-3-acetic acid and cytokinins), phosphate solubilization, iron acquisition, and exopolysaccharide production/biofilm formation. These genomic features are consistent with our *in vitro* analyses, which confirmed high capacity for exopolysaccharide and biofilm production. Moreover, both strains produce indole-3-acetic acid (IAA), are compatible with *Bradyrhizobium japonicum* and *Azospirillum brasilense*, and exhibit antagonistic activity against soilborne phytopathogens, highlighting their biotechnological potential for inoculant development.

## 1 Introduction

Climate change has been reshaping precipitation and temperature patterns worldwide, exerting a drastic impact on tropical agriculture (Rezaei et al., 2023). Several studies indicate a decline in the frequency of rainy days, accompanied by more intense and concentrated precipitation events, resulting in longer and more frequent mid-season dry spells (Medeiros et al., 2022; Feldman et al., 2024; Petrova et al., 2024). This alternation of extremes reduces soil moisture stability, constraining the resilience of tropical agricultural systems (Medeiros et al., 2022).

Brazil is recognized as a global agricultural powerhouse, ranking among the leading producers of several commodities and playing a strategic role in global food security (USDA, 2022). However, historical records and climate projections indicate an expansion of arid and semiarid areas in the country, alongside a contraction of temperate and humid zones, changes that directly impact the geographic distribution of agricultural activities (Dubreuil et al., 2019; de Lima et al., 2023). By 2050, losses in soybean production in Brazil may range from 6 to 37% due to climate change, while for maize these losses may vary from 13 to 29% (Zilli et al., 2020).

An integrated approach merging agronomic practices, genetic improvement, and microbiome engineering is essential to increase crop resilience and mitigate the impacts of drought on agriculture (Franco-Navarro et al., 2025). From an agronomic point of view, conservation techniques such as no-till, crop rotation, the use of cover crops, residue retention, and adjustment of sowing dates have proven effective in improving soil physical structure and enhancing its water-holding capacity (Hermans et al., 2021; Nouri et al., 2021; Muhammad et al., 2024). In plant breeding, advances in high-precision phenotyping coupled with genotyping have accelerated the development of cultivars with deeper root systems, enhanced osmotic adjustment capacity, and higher photosynthetic efficiency under low water availability, thereby stabilizing yields under drought periods (Xiong et al., 2022; Guadarrama-Escobar et al., 2024; He et al., 2024). Moreover, genetic engineering techniques have enabled the targeted introduction or activation of genes associated with drought tolerance (Shelake et al., 2022; Raza et al., 2025).

Plant-associated microorganisms are also recognized an important strategy to mitigate water deficit (Phour and Sindhu, 2022; Ali et al., 2023; El-Saadony et al., 2024). These microorganisms act through multiple mechanisms, including the modulation of phytohormones such as indole-3-acetic acid (IAA), which stimulates root growth, and abscisic acid (ABA), which promotes stomatal closure and reduces water loss through transpiration (El-Saadony et al., 2024). Some microorganisms also produce the enzyme 1-aminocyclopropane-1-carboxylate deaminase (ACC deaminase), which decreases ethylene levels in roots, alleviating the stress (Singh et al., 2023). Additionally, the synthesis of osmoprotectants, volatile organic compounds (VOCs), and exopolysaccharides contribute to osmotic adjustment, preserve membrane integrity, and increase rhizosphere water retention, sustaining plant physiological processes under drought conditions (Naseem et al., 2018; El-Saadony et al., 2024; Gholizadeh et al., 2024).

The composition of plant-associated microbial communities is dynamic, varying throughout plant development whilst also modulated by environmental factors such as water and nutrient availability (Knight et al., 2024). Under water deficit, Gram-positive bacteria, particularly *Bacillus* and *Streptomyces*, tend to predominate due to their desiccation tolerance and adaptive physiological traits (Köberl et al., 2013; Etesami et al., 2023; Gholizadeh et al., 2024; Liu et al., 2024). In this context, bioprospecting and selecting *Bacillus* isolates is a promising strategy for developing inoculants to mitigate water deficit, given their metabolic versatility, environmental resilience, and capacity to promote plant growth (Liu et al., 2025; Mosela et al., 2025). Furthermore, multiple isolates of this genus contribute to nutrient solubilization and the biocontrol of phytopathogens through competition for niches and resources, synthesis of antimicrobial metabolites, and induction of systemic resistance in plants (Fira et al., 2018; Mosela et al., 2022; Pontes et al., 2024), highlighting their ecological multifunctionality.

The present study aimed to bioprospect and select *Bacillus* strains capable of mitigating the effects of water deficit and promoting maize and soybean growth, with a view to developing novel microbial inoculants. To this end, the work was structured in three stages: (i) evaluation of multiple *Bacillus* strains capacity to mitigate water deficit under controlled conditions and across different plant species; (ii) assessment of the selected strains performance over maize and soybean growth under contrasting edaphoclimatic conditions; and (iii) bacterial genomic analysis, including taxonomic identification and detection genes associated with plant growth promotion and mitigation of water deficit.

## 2 Materials and methods

### 2.1 Bacterial strains

Eight *Bacillus* isolates (BAC-01 to BAC-08) from the microbial culture collection of the BIOINPUT company (Paraná, Brazil) were used in this study. The isolates were bioprospected from rhizospheric soil attached to the roots of maize and soybean samples. They were preselected from an *in vitro* screening of a total of 102 isolates based on plant protection and growth-promoting traits.

Additionally, two bacterial-based commercial products were used in this study for comparative purposes, Auras^®^ (*Bacillus aryabhattai* CMAA 1363, NOOA, Minas Gerais, Brazil) and Biotrinsic^®^ (*Bacillus simplex* SYM00260, Indigo, São Paulo, Brazil).

### 2.2 Biomass bioprocess

The isolates stored at −80°C in TSB-glycerol (40% v/v) were activated on LBA medium at 28°C for 24 h. The pre-inoculum for each isolate was prepared from pure colonies suspended in saline solution (0.85% NaCl), adjusted to 0.5 McFarland scale (~1.5 × 10^8^ CFU mL^−1^), and inoculated (0.1% *v/v*) in 30 mL of AgO3 (20 g L^−1^ glucose, 5 g L^−1^ yeast extract, 5 g L^−1^ tryptone, 1 g L^−1^ monobasic potassium phosphate, 0.5 g L^−1^ dibasic potassium phosphate, 0.5 g L^−1^ magnesium sulfate, 0.5 g L^−1^ iron sulfate, 0.5 g L^−1^ calcium chloride, and 0.5 g L^−1^ sodium chloride) medium. The culture was incubated for 18 h at 30°C and 200 rpm. Next, 4 mL of the culture was used to inoculate 400 mL of the same medium, which was then incubated at 30°C with shaking at 200 rpm for 72 h. At the end of the process, the bacterial suspension was standardized to 1.0 × 10^9^ CFU mL^−1^.

### 2.3 Growth chamber experiment

Seeds of *Arabidopsis thaliana* (ecotype Col-0) were surface disinfected in 75% ethanol with 0.1% Triton X-100 for 5 min, rinsed ten times with sterile water, and then kept in the dark at 4°C for three days. The seeds were sown in 80 mL pots containing Carolina Soil^®^ substrate and vermiculite (3:1, *v/v*). Seedlings were grown for 10 days in a growth chamber under a 16:8 light-dark photoperiod at 22°C and then thinned to five per pot. Inoculation was carried out 13 days after sowing by applying 1 mL of the bacterial suspension (1 × 10^9^ CFU mL^-^¹) directly into the soil in proximity to the roots. Treatments consisted of the eight previously selected *Bacillus* isolates, *B. aryabhattai* CMAA 1363, and two uninoculated controls (irrigated and not irrigated). The experimental design was completely randomized with ten replicates, each pot was considered an experimental unit.

For the drought assay, irrigation was suspended at flowering onset, except in the irrigated control. Plants remained without irrigation for 12 days, followed by three days of rehydration. After this period, shoot and root dry mass were determined. The experiment was independently repeated twice.

### 2.4 Greenhouse experiment

The greenhouse experiments were conducted at Centro Universitário Filadélfia (UNIFIL, Londrina, Paraná, Brazil) using soybean (*Glycine max* L.), common bean (*Phaseolus vulgaris* L.), and maize (*Zea mays* L.). The treatment arrangements were identical to those used in the growth chamber assays with *A. thaliana*, arranged in a completely randomized design with six replicates. Each pot was considered an experimental unit.

For soybean, common bean, and maize, the cultivars DM 66I68 IPRO, IPR Sabiá, and Dekalb 360 PRO3 were used, respectively. Seed inoculation was performed at a rate of 100 mL of the bacterial suspension (1 × 10^9^ CFU mL^-^¹) per 50 kg of seed. Soybean and common bean were sown in 5 L pots filled with soil and sand (3:1, *v/v*). Fertilization consisted of applying 3 g of Osmocote^®^ (ICL Specialty Fertilizers, Tel Aviv, Israel; 15% N, 9% P_2_O_5_, 12% K_2_O, 1% Mg, 2.3% S, 0.05% Cu, 0.45% Fe, 0.06% Mn, 0.02% Mo) at sowing and again 30 days after plant emergence. Plants were irrigated to maintain 80% of the substrate’s water-holding capacity (WHC) until the V4 stage. In the water-deficit treatments, irrigation was reduced to 30% WHC for 15 days, beginning at R1 (soybean) and R5 (common bean), followed by rewatering to 80% WHC until physiological maturity. The number of pods per plant, root dry mass, and grain weight per plant were recorded.

For maize, seeds were sown in 10 L pots filled with a 3:1 (*v/v*) mixture of soil and sand. Fertilization consisted of 3 g of Osmocote^®^ at sowing and 100 kg ha^-^¹ of ammonium sulfate applied 20 days after emergence. Irrigation was maintained at 80% WHC until V4. From that point, in the water deficit treatments, irrigation was reduced to 30% WHC for 15 days, followed by rehydration to 80% WHC for 12 days. At the end of this period, shoot and root dry mass were measured.

Soil moisture was monitored with TDR probes (Time Domain Reflectometry, model CS560). Probe calibration was conducted over 25 days using PVC tubes (30 cm height × 10 cm diameter) with bottoms covered in shade cloth and electrical tape to allow drainage without substrate loss.

Additionally, a second maize experiment was conducted with the following treatments: i) irrigated control, ii) non-irrigated control, iii) *Ascophyllum nodosum* (Stingray^®^, Koppert, Netherlands), iv) *Bacillus aryabhattai* CMAA 1363 (1 × 10^8^ CFU mL^-^¹, NOOA), v) *Bacillus simplex* SYM00260 (1 × 10^7^ CFU mL^-^¹, Indigo), vi) *Bacillus* sp. Ag129 (1 × 10^9^ CFU mL^-^¹, BIOINPUT), and vii) *Bacillus* sp. Ag132 (1 × 10^9^ CFU mL^-^¹, BIOINPUT). Growing conditions, irrigation, and experimental design were identical to those described for the first maize experiment.

### 2.5 Field experiment

Field trials were conducted to evaluate the efficacy of biological seed treatments in maize (hybrid Pioneer P3310VYHR) and soybean (cultivar DM 66I68 IPRO). Treatments were: i) control (no inoculation), ii) *A. nodosum* (Stingray^®^, Koppert), iii) *B. aryabhattai* strain CMAA 1363 (1 × 10^8^ CFU mL^-^¹, NOOA), iv) *B. simplex* strain SYM00260 (1 × 10^7^ CFU mL^-^¹, Indigo), v) *Bacillus* sp. Ag129 (1 × 10^9^ CFU mL^-^¹, BIOINPUT), and vi) *Bacillus* sp. Ag132 (1 × 10^9^ CFU mL^-^¹, BIOINPUT).

Seed treatment dosages used were 100 mL per 60,000 maize seeds and 100 mL per 50 kg of soybean seeds, both at 1 × 10^9^ CFU mL^-^¹. The only exception was *B. simplex*, applied at 10 mL per 60,000 maize seeds and 10 mL per 50 kg of soybean seeds at 1 × 10^7^ CFU mL^-^¹. In all soybean trials, seeds were previously inoculated with *Bradyrhizobium japonicum* (strains SEMIA 5079 and 5080) at the standard rate of 100 mL per 50 kg of seeds.

Experiments followed a randomized complete block design with four replicates. Each plot consisted of eight rows, 7 m in length, spaced 0.45 m apart. Before sowing, all areas received 100 kg ha^-^¹ KCl and 20 kg ha^-^¹ nitrogen (urea). For maize, a sidedress application of 138 kg ha^-^¹ nitrogen was made at the V6 stage. Six field trials with maize were conducted at the following locations and seasons: (1) Londrina–PR (2023/2024), (2) Mauá da Serra–PR (2023/2024), (3) Guarapuava–PR (2023/2024), (4) Dourados–MS (2023/2024), (5) Barretos-SP and (6) Rio Verde–GO (2024/2024). For soybean, five trials were carried out: (1) Londrina–PR, (2) Mauá da Serra–PR, (3) Guarapuava–PR, (4) Rio Verde–GO, and (5) Barretos–SP, all in the 2023/2024 growing season. For all areas where data sources were available, water balance was calculated using the Thornthwaite (1948) method. Grain yield (t ha^-^¹) was determined after harvesting the six central rows of each plot. Detailed information on soil physicochemical characteristics and edaphoclimatic conditions at the experimental sites is provided in **Supplementary Table S1**.

### 2.6 Whole genome sequencing and gene prediction

Genomic DNA was extracted from 50 mg of bacterial biomass using the Wizard^®^ gDNA Purification kit (Promega, United States). An aliquot of 1 ng DNA was used to construct the genomic library with the Nextera XT kit (Illumina, United States). Sequencing was performed on an Illumina NextSeq platform with 2 × 300 bp paired end reads (GoGenetic, Curitiba, Brazil).

Raw read quality was assessed using FastQC (Andrews, 2010), and preprocessing was performed with Trimmomatic (Bolger et al., 2014), which applied quality filters and adapter removal. The filtered reads were then re-evaluated with FastQC to ensure sequence integrity and quality. *De novo* genome assembly was performed with MaSuRCA (Zimin et al., 2013), and the resulting contigs were ordered with RagTag (Alonge et al., 2022), using *B. velezensis* MH25 (GenBank: CP034176) as the reference genome.

The genome was functionally annotated using several databases, including the Non-redundant Protein Database, GO, KEGG, COG and Swissprot. To provide a comprehensive overview of the genomic data MGCplotter (https://github.com/moshi4/MGCplotter) was employed. The reference genome was selected based on a preliminary contig annotation with Prokka (Seemann, 2014) and similarity analysis of the 16S *rRNA*, *gyrB*, and *citA* genes using BLASTn (NCBI) to identify the closest species. Genomic similarity to other strains was assessed using OrthoANI (Lee et al., 2016), which included the construction of a UPGMA dendrogram and calculation of the GGDC index. The results were used to generate a heatmap in R with ggplot2 using a custom script. The prediction of genes related to plant growth promotion was performed using the PGPg_Finder program (Pellegrinetti et al., 2024), with default parameters.

### 2.7 *In vitro* characterization of the selected strains

Four bacterial strains were evaluated: *B. simplex* SYM00260, *B. aryabhattai* CMAA 1363, and *B. velezensis* Ag129 and Ag132. Assays were performed under controlled conditions to characterize osmotic stress tolerance, plant growth promotion, and antagonistic activity against phytopathogens.

#### 2.7.1 Osmotic stress tolerance

Tolerance to osmotic stress was assessed on 10% (*w/v*) TSA supplemented with 405 g L^-^¹ sorbitol (water activity = 0.919), as described by Velloso et al. (2020). Strains were streaked and incubated at 30°C for 72 h. Bacterial growth in the plates indicated the ability to thrive under low water activity environment.

#### 2.7.2 Exopolysaccharide production

EPS production was determined as described by Paulo et al. (2012). Standardized bacterial suspensions (0.5 McFarland) were spotted (5 µL) onto sterile filter-paper disks (5 mm diameter) placed on LBA medium. After overnight incubation, mucoid colonies were transferred to 2 mL of absolute ethanol. Precipitate formation indicated positive EPS production. Strains were classified as (−) absent, (+) weak, (++) moderate and (+++) high EPS producers.

#### 2.7.3 Biofilm formation

Biofilm formation was evaluated in 96-well plates according to Stepanović et al. (2007), with adaptations. Cultures grown for 24 h in BHI broth (37°C, 100 rpm) were inoculated (200 µL) in triplicate. After incubation, wells were washed (0.85% NaCl), fixed with methanol (200 µL, 15 min), stained with crystal violet (200 µL, 5 min), and the excess stain was removed. Quantification was performed by resolubilizing the dye with ethanol and measuring absorbance at 570 nm. Classification was based on the ratio between the isolate optical density (OD_i_) and the negative control (ODc). Strains were classified as (−) absent, (+) weak, (++) moderate and (+++) high biofilm producers.

#### 2.7.4 Siderophore production

Siderophore production was assessed using the Chrome Azurol S (CAS) assay (Schwyn and Neilands, 1987), with modifications by Hu and Xu (2011). Strains were grown in 10% TSB for 72 h, and cell-free supernatants (CFS) were mixed 1:1 with CAS reagent. After 20 min, absorbance was read at 630 nm. Production was expressed as percent siderophore units (psu), according to Arora and Verma (2017).

#### 2.7.5 Indole-3-acetic acid production

For IAA quantification, strains were grown in TSB supplemented with 1 g L^-1^ tryptophan for 5 days at 30°C and 100 rpm in the dark. Supernatants were mixed with Salkowski reagent (1:1 mL), incubated for 20 min in the dark, and absorbance was measured at 540 nm and readings were contrasted with an IAA standard curve (Sousa et al., 2021).

#### 2.7.6 Compatibility with commercial strains

Compatibility was evaluated with *B. japonicum* SEMIA 5079 and *Azospirillum brasilense* Ab-V5 using the cross-streak method on YMA or RC agar. After 5–7 days of incubation at 28°C, inhibition zones at the streak intersection indicated inhibition, therefore incompatibility.

#### 2.7.7 Antagonism against phytopathogenic fungi

Antagonistic activity against *Macrophomina phaseolina* and *Sclerotinia sclerotiorum* was evaluated by dual-culture assays on PDA plates. Mycelial plugs were placed at the center of the plate, and bacterial strains were inoculated 1 cm from the plate edges. Plates were incubated at 25°C under a 12-hour light/dark photoperiod for 3–5 days. Inhibition zones around bacterial colonies were taken as evidence of antagonism.

### 2.8 Data analysis

Agronomic data were subjected to analysis of variance and, when the assumptions were met, to Tukey’s multiple comparison test. Data from the growth chamber and greenhouse experiments were analyzed through principal component analysis (PCA) and Ward’s hierarchical clustering based on standardized mean Euclidean distance. For the field data (yield), the (Lin and Binns, 1988) stability index was calculated as follows:


$$P_{i\_a}=[\frac{\sum_{j=1}^{n}{\left(Y_{ij-}Y_{gi}\right)}^{2}}{2n}]\frac{CV_{j}}{CV_{T}}$$

$$P_{i\_f}=[\frac{\sum_{j=1}^{f}{\left(Y_{ij-}Y_{gi}\right)}^{2}}{2f}]\frac{CV_{j}}{CV_{T}}$$

$$P_{i\_u}=[\frac{\sum_{j=1}^{f}{\left(Y_{ij-}Y_{gi}\right)}^{2}}{2u}]\frac{CV_{j}}{CV_{T}}$$

Where: Pi_a ​is the stability statistic defined by Lin and Binns (1988), Pi_f and Pi_u ​​ are the statistics defined by Cruz and Carneiro (2003). “f” and “u” are the numbers of favorable (positive environmental index, including zero, as defined by Eberhart and Russell (1966) and unfavorable (negative environmental index) environments, respectively. n=f+u, $Y_{ij}$ is the phenotypic value of genotype *i* in environment *j*; $Y_{gi}$​ is the ideal response of a hypothetical genotype in environment *j* estimated by the two-segment model of Cruz et al. (1989). $CV_{j}$ and $CV_{T}$​ correspond to the residual coefficient of variation for environment *j* and the sum of the coefficients of variation across all environments, respectively. All analyses were performed in R using the packages metan (Olivoto and Lúcio, 2020), AgroR (Shimizu et al., 2025), and FactoMineR (Lê et al., 2008).

## 3 Results

### 3.1 Growth chamber experiment with *Arabidopsis thaliana*

Analysis of variance revealed that treatments significantly influenced both root dry mass (RDM) and shoot dry mass (SDM) in Experiment I with *A. thaliana*. In contrast, in Experiment II, significant effects were observed only for SDM (**Table 1**). For RDM, a reduction of 44% was detected between the irrigated and water-deficit controls, evidencing the impact of drought on root development. In Experiment I, strains CMAA 1363, BAC-02, BAC-04, BAC-06, BAC-07, and BAC-08 did not differ from the irrigated control in terms of RDM. For SDM, reductions of 27% and 34% were observed in Experiments I and II, respectively, when comparing the irrigated vs. water-deficit controls. In Experiment I, treatments BAC-04 and BAC-08 stood out, whereas in Experiment II, strain BAC-04 maintained an superior performance than the water-deficit control.

**Table 1:** Tukey’s multiple comparison test and significance analysis of treatments inoculated with *Bacillus* sp. in water deficit experiments with *Arabidopsis thaliana* under growth chamber conditions.

Treatments	Experiment I		Experiment II	
	RDM^1/^	SDM	RDM	SDM
Control (irrigated)	0.092 a	0.371 a	0.038 a	0.421 a
Control (water deficit)	0.052 c	0.271 b-d	0.034 a	0.279 b
CMAA 1363	0.075 a-c	0.205 d	0.042 a	0.269 b
BAC-01	0.065 bc	0.259 cd	0.040 a	0.259 b
BAC-02	0.082 ab	0.296 bc	0.034 a	0.296 b
BAC-03	0.054 c	0.257 cd	0.032 a	0.272 b
BAC-04	0.085 ab	0.316 a-c	0.025 a	0.320 ab
BAC-05	0.062 bc	0.271 b-d	0.033 a	0.280 b
BAC-06	0.074 a-c	0.294 bc	0.032 a	0.295 b
BAC-07	0.068 a-c	0.254 cd	0.030 a	0.265 b
BAC-08	0.077 a-c	0.331 ab	0.038 a	0.298 b
p-value (treatments)^2/^	0.040	0.002	0.480	<0.001

^1/^RDM, root dry mass (g); SDM, shoot dry mass (g).

^2/^Means followed by the same letter in the same column do not differ significantly by Tukey’s test at the 5% probability level.

### 3.2 Greenhouse experiment with soybean, maize, and common bean

In the greenhouse experiment with common bean, water deficit caused reductions of 25, 32, and 48% in RDM, number of pods per plant (NPP), and grain dry mass per plant (GDM), respectively, compared with the irrigated control (**Table 2**). For RDM, the BAC-06 treatment performed statistically on par with the irrigated control. Similarly, for NPP, the same was observed for BAC-04, BAC-06, BAC-07, and BAC-08. For GDM, BAC-04 and BAC-05 presented the highest values under water deficit, surpassing the non-irrigated control by 17.8% and 14.8%, respectively. In addition, BAC-01, BAC-02, BAC-06, BAC-07, and BAC-08 were statistically similar to BAC-04 and BAC-05.

**Table 2:** Tukey’s multiple comparison test and significance analysis of treatments inoculated with *Bacillus* sp. in water deficit experiments with common bean, soybean, and maize under greenhouse conditions.

Tratamentos	Common bean			Soybean			Maize	
	RDM^1/^	NPP	GDM	RDM	NPP	GDM	RDM	SDM
Control (irrigated)	3.89 a	33.17 a	48.83 a	12.69 a-c	62.17 a	37.84 a	26.56 a	58.03 a
Control (water deficit)	2.91 b-d	22.71 b	25.57 c	9.51 d	26.40 de	26.48 c	12.75 c	43.61 b
*B. aryabhattai*	2.63 b-d	23.50 b	25.58 c	11.70 b-d	41.33 bc	28.67 bc	12.82 c	47.61 ab
BAC-01	3.10 bc	23.40 b	26.64 bc	13.30 ab	36.20 b-d	26.84 c	12.79 c	43.42 b
BAC-02	2.81 b-d	21.83 b	28.08 bc	13.60 ab	36.83 b-d	32.2 b	13.45 c	42.88 b
BAC-03	2.56 cd	18.33 b	24.79 c	12.31 ab	44.83 b	36.96 ab	14.33 bc	46.59 ab
BAC-04	2.82 b-d	25.75 ab	30.13 b	14.79 a	44.67 b	33.37 bc	19.91 b	49.62 ab
BAC-05	2.92 b-d	21.67 b	29.35 b	10.21 cd	39.75 bc	21.52 d	16.79 bc	49.10 ab
BAC-06	3.29 ab	25.17 ab	26.90 bc	12.49 a-c	19.84 e	24.97 cd	16.72 bc	45.84 ab
BAC-07	2.28 d	25.83 ab	26.78 bc	12.71 a-c	41.50 bc	31.17 bc	16.99 bc	46.11 ab
BAC-08	2.82 b-d	24.17 ab	26.68 bc	11.79 b-d	33.80 cd	27.74 c	11.98 c	43.76 b
p-value (treatments)^2/^	<0.001	<0.001	<0.001	0.04	<0.001	0.03	<0.001	0.03

^1/^RDM, grain dry mass (g); NPP, number of pods per plant; GDM, grain dry mass per plant (g); SDM, shoot dry mass.

^2/^Means followed by the same letter in the same column do not differ statistically by Tukey’s test at the 5% probability level.

In soybean, water deficit significantly reduced RDM, NPP, and GDM by 25, 58, and 30%, respectively, compared with the irrigated control. For RDM, treatments with BAC-01, BAC-02, BAC-03, BAC-04, BAC-06, and BAC-07 did not differ statistically from the irrigated control and showed increases over the water-deficit control of 39.9, 43.0, 29.4, 55.4, 31.3, and 33.6%, respectively. For NPP, CMAA 1363, BAC-03, BAC-04, BAC-05, and BAC-07 produced marked increases relative to the water-deficit control, with gains of 56.5, 69.8, 69.2, 50.6, and 57.2%, respectively. For GDM, BAC-02 and BAC-03 recorded the highest values under water deficit, without statistical differences from CMAA 1363, BAC-04, and BAC-07.

In maize, water deficit significantly reduced RDM and SDM by 52 and 25%, respectively, compared with the irrigated control. Under the stress condition, BAC-04 recorded the highest RDM, with a 56.2% increase relative to the non-irrigated control. However, its performance did not differ statistically from BAC-03, BAC-05, BAC-06, and BAC-07. Regarding SDM, CMAA 1363, BAC-03, BAC-04, BAC-05, BAC-06, and BAC-07, were no statistically different from the irrigated control.

**Figure 1:**
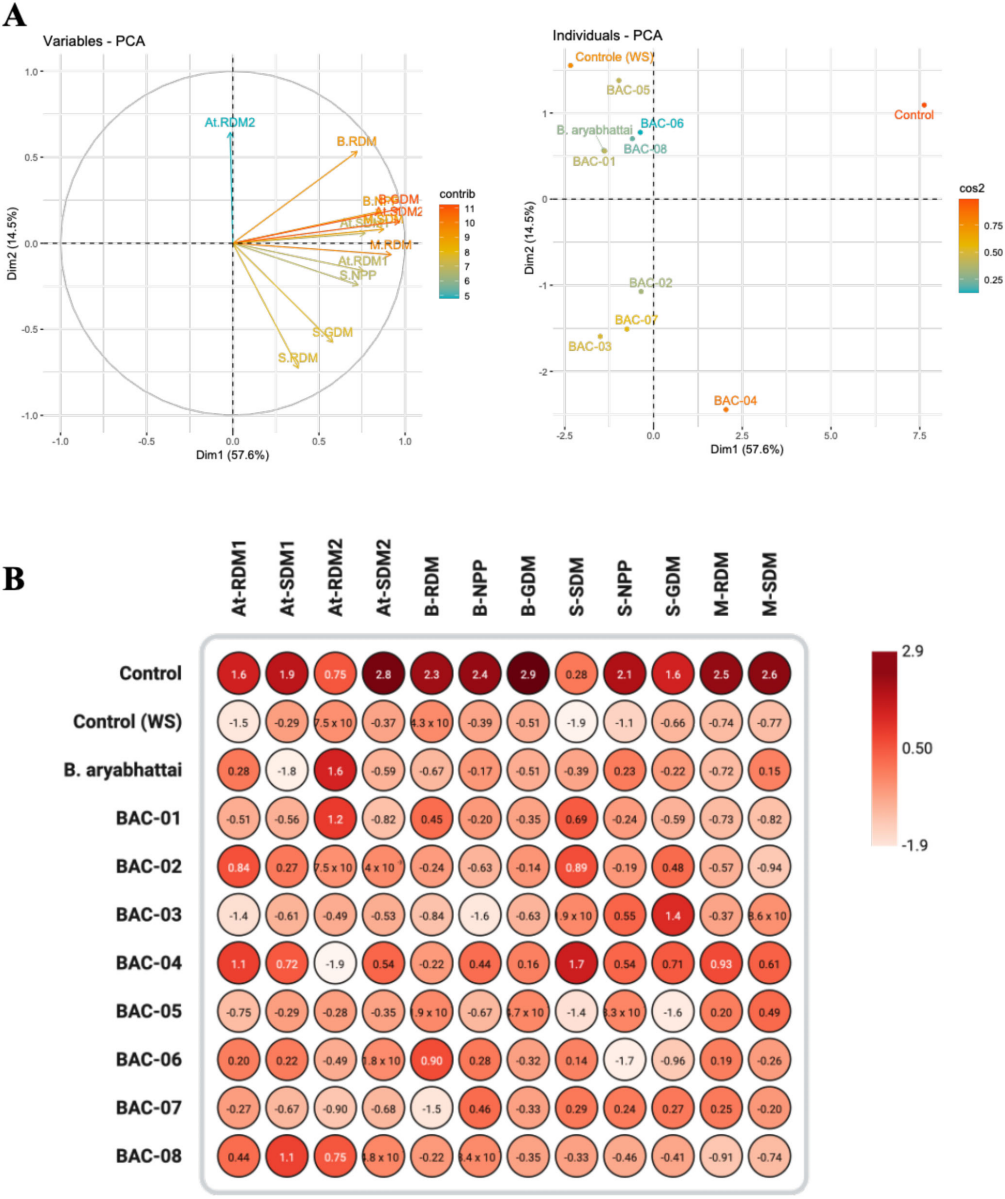
**(A)** Principal component analysis and **(B)** heatmap for the evaluation of agronomic traits in treatments inoculated with *Bacillus* sp. under water deficit experiments in *Arabidopsis thaliana* (At), common bean **(B)**, soybean (S), and maize (M). RDM, root dry mass; SDM, shoot dry mass; NPP, number of pods per plant; GDM, grain dry mass per plant.

Principal component analysis (PCA) indicated that the first two components explained 72.1% of the total variation (PC1 = 57.6% and PC2 = 14.5%) (**Figure 1A**). RDM, SDM, NPP, and GDM loaded strongly and positively on PC1, making them the principal discriminating variables among treatments. The biplot revealed distinct clustering patterns, with BAC-04 and the irrigated control clearly separated from the other treatments and positioned near to the vectors of the variables, indicating superior agronomic performance. BAC-02, BAC-03, and BAC-07 occupied an intermediate region of the biplot, while the remaining treatments were more dispersed and tended toward negative scores for the evaluated variables. These trends were corroborated by the heatmap (**Figure 1B**), which highlighted the favorable multivariate profiles of BAC-04 and BAC-07. Both isolates were selected for subsequent stages and renamed as Ag129 and Ag132, respectively.

**Figure 2:**
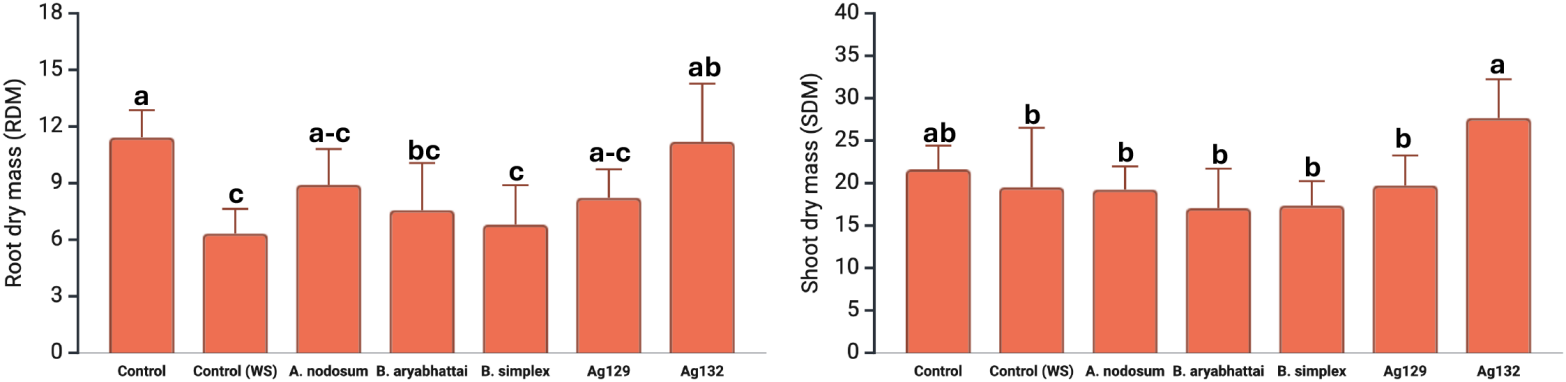
Effect of *Bacillus* inoculation and *Ascophyllum nodosum* extract on maize growth under greenhouse water-deficit conditions. Root and shoot dry mass (RDM and SDM) are shown for each treatment. Bars represent means ± SE (n = 6). Different letters indicate significant differences among treatments by Tukey’s HSD test (p < 0.05).

In the second maize experiment, significant treatment effects were observed for RDM and SDM. For RDM, water deficit resulted in a 50% reduction compared with the irrigated control (**Figure 2**). The *Ascophyllum nodosum*, Ag129, and Ag132 treatments achieved RDM values statistically similar to the irrigated control. For SDM, the highest value was obtained with strain Ag132, which showed a 41.9% increase relative to the water-deficit control and did not differ statistically from the irrigated control.

### 3.3 Field experiments with maize and soybean

In the maize trials, analysis of variance indicated that yield was significant influenced by all sources of variation, including treatments (T), environments (A), and the T×A interaction (**Table 3**). The coefficient of variation (CV) was 10.52%, reflecting good data precision. Based on overall means, most treatments increased yield compared with the untreated control, except for strain CMAA 1363. The greatest average gain was obtained with strain SYM00260 (14.38%), followed by Ag129 (10.01%), Ag132 (8.79%), and the *A. nodosum* extract (6.91%).

**Table 3:** Analysis of variance (ANOVA) for maize and soybean grain yield (t ha^-^¹) in multi-environment field trials with biological seed treatments.

Source of variation	Maize		Soybean	
	DF	Mean square	DF	Mean square
Repetitions/E	18	0.610	15	0.117
Treatments (T)	5	485.18 (p<0.001)	5	42.140 (p<0.001)
Environment (E)	5	3.81 (p<0.001)	4	1.036 (p<0.001)
T x E	25	1.06 (p<0.011)	20	0.331 (p<0.003)
Error	90	0.57	75	0.14
CV (%)	10.52		10.26	
Mean				
Control	6.75		3.42	
*Ascophyllum nodosum*	7.22 (**6.91%**)^1/^		3.45 (**0.88%**)	
*B. aryabatthai* CMAA 1363	6.71 (**-0.71%**)		3.75 (**9.65%**)	
*B. simplex* SYM00260	7.72 (**14.38%**)		3.41 (-**0.29%**)	
*B. velezensis* Ag129	7.43 (**10.01%**)		3.79 (**10.82%**)	
*B. velezensis* Ag132	7.35 (**8.79%**)		3.94 (**15.20%**)	

1/ Percentage increase in yield relative to the control.

Evaluation of yield by environment revealed that, in most locations, the use of biological products increased yield compared with the control (**Table 4**). The exception was Mauá da Serra (2023/2024 season), where no significant differences were detected among treatments. Treatments with SYM00260, Ag129, Ag132, and *A. nodosum* extract promoted yield gains in most environments, reflected in low values of the Lin and Binns superiority index for broad environments (Pi_a), with SYM00260, Ag129, and Ag132 standing out. For the superiority index in favorable environments (Pi_f), the lowest value was observed for SYM00260, followed by Ag132 and Ag129, indicating high performance under those conditions. In unfavorable environments (Pi_u), Ag129 had the best performance, followed by SYM00260, *A. nodosum*, and Ag132, evidencing the stability of these treatments under contrasting edaphoclimatic conditions.

**Table 4:** Maize grain yield (t ha^-^¹) across six field environments and yield stability of biological seed treatments based on Lin and Binns’ superiority index.

Treatments	Yield (t ha^-1^)^1/^						Lin e Binns’ superiority index^3/^		
	Env1^2/^	Env2	Env3	Env4	Env5	Env6	Pi_a	Pi_f	Pi_u
Control	14.97 bc	4.97 a	8.97 b	5.05 b	2.54 b	4.00 c	0.85	1.12	0.71
*Ascophyllum nodosum*	13.91 c	4.48 a	10.66 a	5.84 ab	3.40 a	5.01 a	0.49	1.10	0.19
*B. aryabatthai* CMAA 1363	14.10 bc	4.19 a	9.88 ab	5.07 b	2.59 b	4.40 b	0.84	1.13	0.70
*B. simplex* SYM00260	16.01 a	5.19 a	10.82 a	5.89 ab	3.08 a	5.34 a	0.08	0.00	0.12
*B. velezensis* Ag129	15.57 a	4.36 a	9.74 ab	6.56 a	3.40 a	4.94 ab	0.19	0.34	0.11
*B. velezensis* Ag132	15.10 ab	4.70 a	10.33 ab	6.81 a	2.25 b	4.87 ab	0.23	0.26	0.22

^1/^Env1: Londrina (23/24), Env2: Mauá da Serra (23/24), Env3: Guarapuava (23/24), Env4: Dourados (23/24), Env5: Barretos (23/24), and Env6: Rio Verde (24/24).

^2/^Means followed by the same letter in the column do not differ statistically by Tukey’s method (p < 0.10).

^3/^Pi_a: superiority index across all environments; Pi_f: superiority index in favorable environments; Pi_u: superiority index in unfavorable environments.

In the soybean trials, a significant yield effect was also observed for all sources of variation (CV = 10.26%) (**Table 3**). Based on overall means, most treatments increased yield compared with the untreated control, except for strain SYM00260 and the *A. nodosum* extract. The highest average gain was recorded with strain Ag132 (15.32%), followed by Ag129 (10.82%), and CMAA 1363 (9.65%).

Analysis of yield by environment in soybean showed that, in most locations, the biological treatments with strains CMAA 1363, Ag129, and Ag132 increased yield relative to the control (**Table 5**). The only exception was Barretos (2023/2024 season), where no significant differences among treatments were detected. Based on Pi_a and Pi_f, strain Ag132 had the lowest values, indicating the most stable and productive performance under those conditions, followed by Ag129 and CMAA 1363. For Pi_u, the lowest value was observed for CMAA 1363, followed by *A. nodosum*, Ag129, and Ag132, suggesting greater effectiveness of these treatments under adverse growing conditions.

**Table 5:** Soybean grain yield (t ha^-^¹) across five field environments and yield stability of biological seed treatments based on Lin and Binns’ superiority index.

Treatments	Yield (t ha^-1^)^1/^					Lin e Binns’ superiority Index^3/^		
	Env1^2/^	Env2	Env3	Env4	Env5	Pi_a	Pi_f	Pi_u
Control	3.98 bc	5.29 bc	3.18 bc	2.82 b	1.80 a	0.23	0.37	0.14
*Ascophyllum nodosum*	3.90 c	4.72 c	3.66 a	3.30 a	1.67 a	0.31	0.74	0.02
*B. aryabatthai* CMAA 1363	4.52 a	5.24 bc	3.90 a	3.22 a	1.88 a	0.10	0.24	0.00
*B. simplex* SYM00260	4.47 ab	4.98 c	3.01 c	2.69 b	1.91 a	0.28	0.38	0.21
*B. velezensis* Ag129	4.43 ab	5.72 ab	3.60 ab	3.38 a	1.80 a	0.05	0.09	0.02
*B. velezensis* Ag132	4.84 a	6.16 a	3.50 ab	3.27 a	1.90 a	0.02	0.00	0.03

^1/^Env1: Londrina (23/24), Env2: Mauá da Serra (23/24), Env3: Guarapuava (23/24), Env4: Rio Verde (23/24), and Env5: Barretos (23/24).

^2/^Means followed by the same letter in the column do not differ statistically by Tukey’s method (p < 0.10).

^3/^Pi_a: superiority index across all environments; Pi_f: superiority index in favorable environments; Pi_u: superiority index in unfavorable environments.

### 3.4 Genomic analysis – strains Ag129 and Ag132

**Figure 3:**
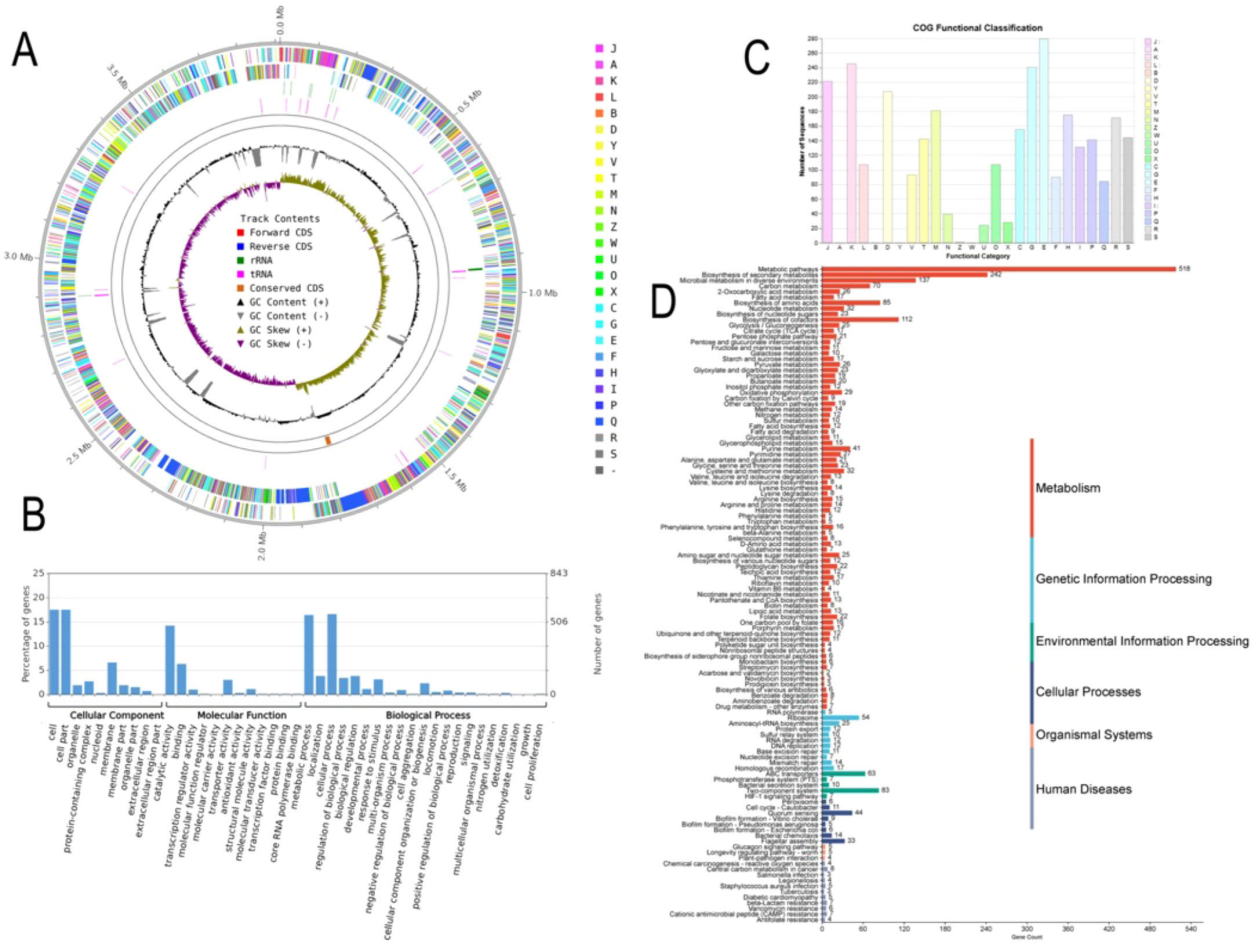
Database annotation for *B. velezensis* Ag129. **(A)** A circular genome map is presented, showing the scale; GC skew; GC content; COG classifications for coding DNA sequences (CDS); and the specific positions of CDS, transfer RNA (tRNA), and ribosomal RNA (rRNA) on the genome. This map offers a comprehensive overview of the genomic structure. **(B)** GO database annotation, **(C)** COG database annotation, and **(D)** KEGG database annotation.

**Figure 4:**
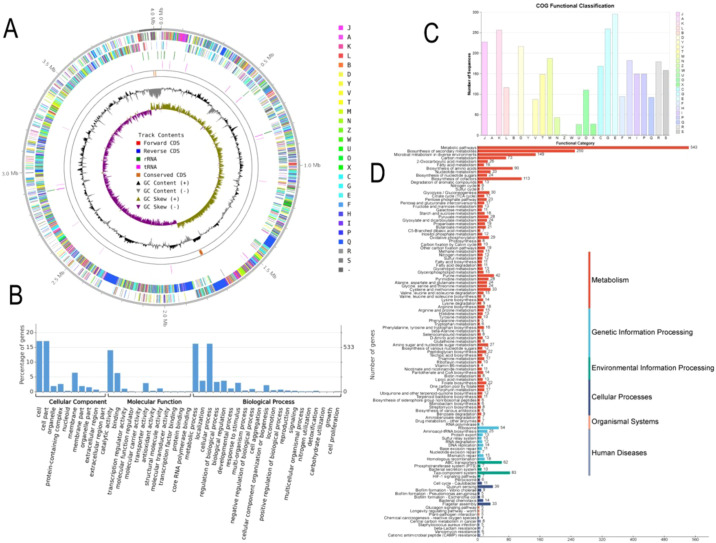
Database annotation for *B. velezensis* Ag132. **(A)** A circular genome map is presented, showing the scale; GC skew; GC content; COG classifications for coding DNA sequences (CDS); and the specific positions of CDS, transfer RNA (tRNA), and ribosomal RNA (rRNA) on the genome. This map offers a comprehensive overview of the genomic structure. **(B)** GO database annotation, **(C)** COG database annotation, and **(D)** KEGG database annotation.

Based on the genome assembly, strains Ag129 (GenBank accession number: SAMN53034369; culture collection: CCT8139) and Ag132 (GenBank accession number: SAMN53034370; culture collection: CCT8141) were identified as *Bacillus velezensis*. The assembled genomes measured 4,046,556 bp and 4,039,722 bp, with alignment rates of 98.20 and 98.14% and average guanine–cytosine (GC) contents of 46.14 and 46.22%, respectively (**Figures 3A**, **4A**). Functional analysis of the genomic sequences was performed using the Cluster of Orthologous Groups of Proteins (COG), Gene Ontology (GO), and Kyoto Encyclopedia of Genes and Genomes (KEGG) databases. The distribution of genes in these databases is shown in **Figures 3**, **4** for strains Ag129 and Ag132, respectively.

Of the 3,005 and 3,169 genes identified in the genomes of strains Ag129 and Ag132, respectively, 2,861 and 3,011 were assigned to 22 COG functional categories. The remaining 144 and 158 genes were classified as S (unknown function). The most represented classes were E (amino acid transport and metabolism), G (carbohydrate transport and metabolism), K (transcription), and J (translation, ribosomal structure, and biogenesis).

Based on the GO annotation data, 1,011 and 1,050 genes were identified for strains Ag129 and Ag132, respectively. Among these, binding functions accounted for 214 and 223 genes, while cell motility and locomotion were represented by 10 and 17 genes in Ag129 and Ag132, respectively. KEGG mapping classified 2,259 and 2,369 genes into 252 and 265 pathways for Ag129 and Ag132, respectively. For both strains, the most representative categories were metabolic pathways, biosynthesis of secondary metabolites, biosynthesis of antibiotics, microbial metabolism in diverse environments, and ABC transporters. Taken together, these data indicate a broad metabolic repertoire coupled to signaling and transport modules commonly associated with environmental sensing, resource acquisition, biofilm formation, and colonization of plant tissues.

**Figure 5:**
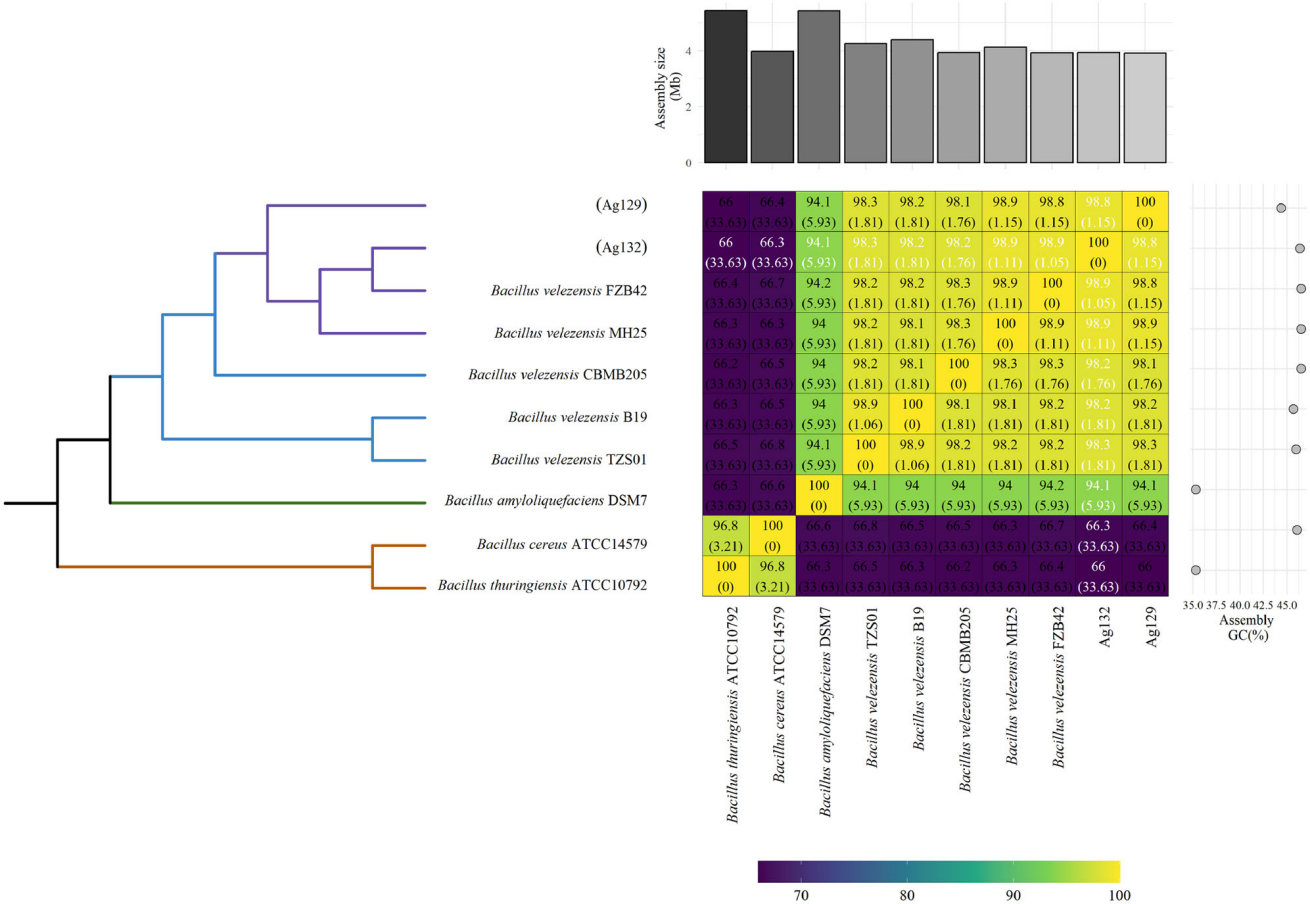
Phylogenetic dendrogram based on maximum likelihood analysis using ten available genome assemblies of *Bacillus velezensis*, *B. amyloliquefaciens*, *B. cereus*, and *B. thuringiensis*, with annotations in a heatmap. Average Nucleotide Identity (ANI, %) values are shown in the heatmap, ranging from the lowest sequence identity (violet) to the highest (green to yellow), grouped according to the phylogenetic tree. The heatmap is annotated with a bar chart displaying the different sizes (Mb) of the ten assemblies (at the top) and their respective GC contents (%) (on the right).

Genomic similarity analysis based on the OrthoANI index revealed high nucleotide identity between strains Ag129 and Ag132 and other *B. velezensis* reference strains (**Figure 5**). Both showed ANI values above 98%, confirming their taxonomic assignment within *B. velezensis*. The dendrogram constructed using UPGMA clustering demonstrated close phylogenomic relatedness between Ag129 and Ag132, which formed a monophyletic clade with *B. velezensis* strains previously described as plant growth-promoting and biocontrol agents.

**Figure 6:**
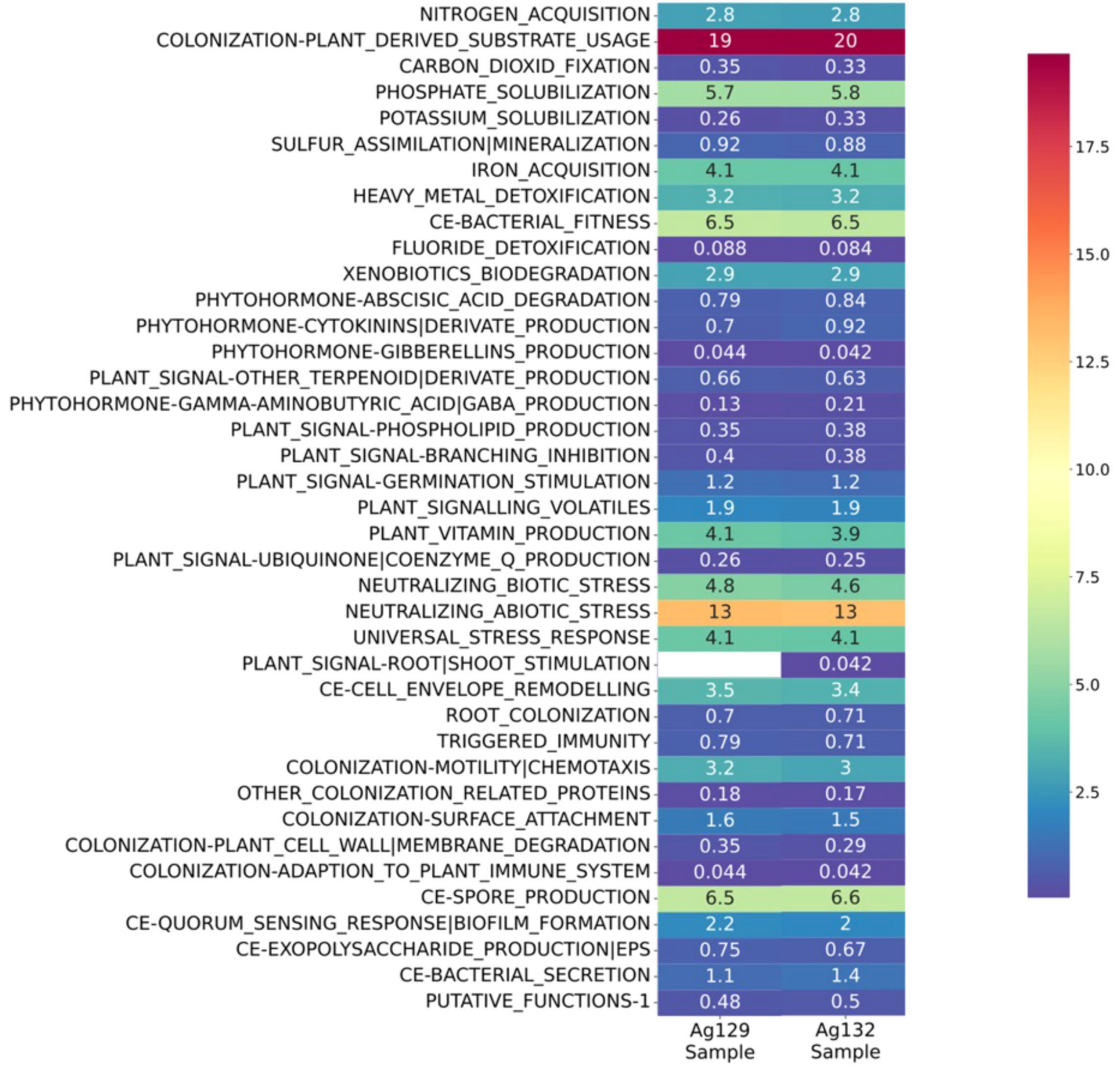
Heatmap of the distribution of genes related to traits that promote plant growth in the genomic sequences of *Bacillus velezensis* strains Ag129 and Ag132.

The functional annotation of the genomes of strains Ag129 and Ag132 revealed a broad diversity of genes associated with plant growth–promoting traits (PGPTs) (**Figure 6**). Both strains harbored genes related to abiotic stress mitigation, phytohormone production, phosphate solubilization, iron acquisition, and biofilm formation, attributes important for performance under water-deficit conditions. Strain Ag129 showed a higher content of genes linked to the universal stress response, rhizosphere colonization, and the metabolism of organic compounds. By contrast, Ag132 was distinguished by an enriched functional set of genes associated with exopolysaccharide synthesis, cytokinin production, and antioxidant mechanisms.

### 3.5 *In vitro* analysis

Based on *in vitro* analysis, strains CMAA1363, Ag129, and Ag132 were able to grow under osmotic stress, indicating tolerance to water-deficit conditions (**Table 6**). In the assessment of EPS and biofilm production, strains Ag129 and Ag132 were classified as strong producers (++++), whereas SYM00260 and CMAA1363 showed weak to moderate production (+/++), suggesting a lower capacity for aggregation and adhesion. Regarding the production of siderophores and IAA, strain CMAA1363 showed the highest values, followed by SYM00260, Ag129, and Ag132. All strains exhibited compatibility with the commercial strains *A. brasilense* Ab-V5 and *B. japonicum* SEMIA 5079, indicating feasibility for co-inoculation. In antagonism assays against phytopathogens, strains Ag129 and Ag132 inhibited the growth of *R. solani* and *M. phaseolina*, demonstrating additional potential for use in biocontrol strategies.

**Table 6:** Plant growth promotion and antagonism traits against phytopathogenic fungi of the strains *Bacillus simplex* SYM00260, *B. aryabattai* CMAA 1363, *B. velezensis* Ag129, and *B. velezensis* Ag132.

Characteristics	*Bacillus*			
	SYM00260	CMAA 1363	Ag129	Ag132
Drought stress tolerance	–	+	+	+
EPS production	+	++	+++	+++
Biofilm production	+	+	+++	+++
Siderophore production at 72h (psu)	29.1	42.3	17.2	27.1
IAA production (µg mL**^-1^**)	25.04	34.93	11.09	8.57
Compatibility with Ab-V5	+	+	+	+
Compatibility with SEMIA 5079	+	+	+	+
Antagonism against *Ralstonia solani*	–	–	+	+
Antagonism against *Macrophomina phaseolina*	–	–	+	+

(+) and (−) indicate positive and negative for the evaluated traits, respectively; for EPS and biofilm production, (+) denotes a weak producer, (++) a moderate producer, and (+++) a strong producer.

## 4 Discussion

The use of *Bacillus* strains has become an important strategy for developing inoculants to mitigate the effects of water deficit in diverse crops (Rashid et al., 2022; Liu et al., 2023; Rajkumar et al., 2024). In the present study, *B. velezensis* strains Ag129 and Ag132 were effective in promoting plant growth under drought conditions across multiple hosts, including *A. thaliana*, common bean, maize, and soybean, reinforcing their broad functionality. Their consistent performance across different plant species highlights their versatility and potential as probiotics for agricultural use, given that the effectiveness of plant growth–promoting microorganisms can be influenced by factors such as the host plant species, the crop’s phenological stage, and environmental conditions (Drogue et al., 2012; Stoll et al., 2021; Kaleh et al., 2025; Taheri et al., 2025). Several studies have indicated the potential of *B. velezensis* under water-deficit conditions, highlighting its effectiveness in soybean (Kondo et al., 2025), common bean (Zamani et al., 2024), rice (Park et al., 2024), and alfalfa (Yin et al., 2025).

*B. velezensis* employs multiple mechanisms that modulate plant gene expression under drought conditions, acting mainly through hormonal regulation, osmotic adjustment, induction of antioxidant enzymes, and enchanced water uptake. Park et al. (2024) demonstrated that inoculation with strain GH1–13 in rice increased drought tolerance by activating genes associated with antioxidant responses and jasmonic acid–mediated signaling. Similarly, strain G138, isolated from arid soils, enhanced water resilience in alfalfa and *A. thaliana* by stimulating the accumulation of osmolytes such as proline and soluble sugars, in addition to inducing the expression of genes related to the drought-stress response (Yin et al., 2025). In soybean, strain S141 significantly increased RDM under drought, likely trough phytohormone-mediated stimulation of root growth (Kondo et al., 2025).

Inoculation with biological products contributed to greater yield stability in maize and soybean under field conditions. In maize, the *B. simplex* (SYM00260) and *B. velezensis* (Ag129 and Ag132) strains stood out for their higher stability and productivity. Nawaz et al. (2024) showed that inoculation with *B. simplex* increased RDM, SDM, and water-use efficiency under water-deficit conditions, highlighting this species’ potential to mitigate drought effects. In Brazil, Senger et al. (2022) reported a 24% increase in maize yield following inoculation with *B. simplex*.

For soybean, the best performance in terms of yield stability and field yield increase was observed for the strains *B. aryabattai* (CMAA 1363) and *B. velezensis* (Ag129 and Ag132), corroborating the rhizosphere-colonization capacity and growth promotion associated with *B. velezensis*. By contrast, the inconsistent performance of *B. simplex* in soybean may be related to strain–host specific interactions, as well as the influence of environmental factors.

The *B. aryabattai* strain CMAA 1363 has been widely investigated and used in Brazil as an inoculant with the potential to mitigate the effects of water deficit in maize (Fuga et al., 2023; Souza et al., 2025). However, in this study, the strain did not increase maize yield, which may be attributed to genotype-dependent interactions or to the influence of specific environmental conditions affecting the inoculant performance. Zeffa et al. (2020) reported differential responses of maize genotypes to inoculation with *A. brasilense*, indicating a genotype-dependent response.

The effectiveness of *B. velezensis* strains (Ag129 and Ag132) in promoting maize and soybean growth may be related to their genetic repertoire of plant growth–associated genes. The presence of genes involved in phytohormone synthesis (IAA and ABA), phosphate solubilization, iron acquisition, and biofilm formation represents key mechanisms for stimulating root growth and supporting microbial adaptation and persistence in the rhizosphere (El-Saadony et al., 2024). These results were corroborated by the *in vitro* analyses, which demonstrated these strains’ ability to grow under osmotic stress and produce high levels of EPS and biofilm. By contrast, these strains showed the lowest IAA and siderophore production values; notably, strain CMAA1363 exhibited the highest levels.

The compatibility of strains Ag129 and Ag132 with commercial strains of *B. japonicum* and *A. brasilense* enables the exploration of synergistic effects between microorganisms with complementary functions, enhancing the physiological and biochemical benefits to the host plant (Bargaz et al., 2021; Wang et al., 2022). This compatibility is strategic for developing efficient microbial consortia, especially in crops such as maize and soybean. In addition, both strains exhibited antagonistic activity against important soilborne phytopathogenic fungi, underscoring their multifunctional potential. Collectively, these results highlight the biotechnological potential of Ag129 and Ag132 as inoculants for promoting plant growth and mitigating the effects of water deficit in tropical agricultural systems.

## 5 Conclusion

Using an integrated approach combining functional screening, phenotypic validation across multiple experimental systems, and genomic analysis, this study identified two *B. velezensis* strains (Ag129 and Ag132) with biotechnological potential as inoculants in crops under water-deficit conditions. Both strains demonstrated the ability to promote the growth of different plant species under drought stress and showed agronomic stability across diverse environments for maize and soybean. Additionally, their compatibility with *B. japonicum* and *A. brasilense*, together with the antagonistic activity against soilborne phytopathogens, highlights the potential of these strains for multifunctional formulations and microbial consortia.

## Data Availability

The original contributions presented in the study are publicly available. This data can be found here: JBUAPR000000000.1 (Ag129) and JBUAPQ000000000.1 (Ag132).
