# Supplementary material for: Bacillus velezensis Ag129 and Ag132: two novel probiotics enhancing drought tolerance and agronomic performance in maize and soybean
Source: Front Plant Sci. 2026 Feb 13;17:1711687. doi: 10.3389/fpls.2026.1711687 (PMC12946138; doi:10.3389/fpls.2026.1711687)
Supplement: Supplementary file 1 [file SupplementaryFile1.docx]

***Bacillus velezensis* Ag129 and Ag132: two novel probiotics enhancing drought tolerance and agronomic performance in maize and soybean**

Antoni Wallace Marcos^1^, Juarez Pires Tomaz^2^, Alison Fernando Nogueira^1^, Mirela Mosela^3^, Daniel Soares Alves^4^, José dos Santos Neto^4^, Lycio Shinji Watanabe^5^, Leandro Afonso^3^, Marcos Ventura Faria^6^, Liliane Scislowski^6^, Daniel Fernando Viana Fagundes^1^, Henry Boguschi Cava^1^, Pablo Diego Silva Cabral^7^, Roger Wisniewski da Conceição^7^, Rafael de Assis^1^, Sérgio Vicente de Azevedo^8^, Liliam Silvia Candido^9^ and Leandro Simões Azeredo Gonçalves^1*^

^1^Agronomy Department, Universidade Estadual de Londrina (UEL), Londrina, Paraná, 86057-970, Brazil

^2^Plant Breeding and Propagation Area, Instituto Rural do Paraná (IDR-Paraná), Londrina, Paraná, 86047-902, Brazil

^3^Microbiology Department, Universidade Estadual de Londrina (UEL), Londrina, Paraná, 86057-970, Brazil

^4^Agronomy Department, Centro Universitário Filadélfia (UNIFIL), Londrina, Paraná, 86045-769, Brazil

^5^Chemical Departament, Universidade Estadual de Londrina (UEL), Londrina, Paraná, 86057-970, Brazil

^6^Agronomy Department, Universidade Estadual do Centro Oeste (UNICENTRO), Guarapuava, Paraná, 85040-167, Brazil

^7^Agronomy Department, Instituto Federal Goiano (IFG), Rio Verde, Goiás, 75901-970, Brazil

^8^Biology Department, Instituto Federal de São Paulo (IFSP), Barretos, São Paulo, 14781-502, Brazil

^9^Biology Department, Universidade Federal de Grande Dourados (UFGD), Dourados, Mato Grosso do Sul, 79804-970, Brazil

*Corresponding author: [leandrosag@uel.br](mailto:leandrosag@uel.br)

**Supporting information**

Additional supporting information may be found in the online version of this article.

**Table S1**.

Characterization of the soil used in the greenhouse experiments

| Characteristics | Experiments | | |
| --- | --- | --- | --- |
|  | Common bean | Soybean | Maize |
| Soil | Dystroferric Red Nitisol | Dystroferric Red Nitisol | Dystroferric Red Nitisol |
| pH(CaCl_2_) | 5.45 | 5.33 | 5.6 |
| H+Al (cmolc dm^3^) | 3.22 | 3.33 | 3.14 |
| K (cmolc dm^3^) | 0.83 | 0.85 | 0.90 |
| Ca (cmolc dm^3^) | 5.82 | 5.50 | 5.60 |
| Mg (cmolc dm^3^) | 2.5 | 2.2 | 2.7 |
| Al (cmolc dm^3^) | 0.0 | 0.0 | 0.0 |
| P (cmolc dm^3^) | 12.23 | 14.01 | 12.77 |
| Organic Matter (%) | 8.55 | 7.92 | 8.22 |

**Tabela S2.**

Characterization of environments used in maize and soybean experiments.

| **Characteristics^1/^** | **Londrina - PR** | **Guarapuava - PR** | **Mauá da Serra - PR** | **Dourados - MS** | **Barretos – SP** | **Rio Verde - GO** |
| --- | --- | --- | --- | --- | --- | --- |
| Geographical coordinates | 23^o^ 17`S; 51^o^ 10`W | 25^o^ 23`S; 51^o^ 29`W | 25^o^ 23`S; 51^o^ 29`W | 22^o^ 13`S; 54^o^ 59`W | 20^o^ 30`S; 48^o^ 33`W | 17^o^ 48`S; 50^o^ 54`W |
| Altitude (m) | 550 | 1026 | 895 | 409 | 565 | 745 |
| Climate^2/^ | Cfa | Cfb | Cfb | Am | Aw | Aw |
| Soil | Dystroferric Red Nitisol | Dystrophic Brown Latosol | Dystrophic Red Latosol | Dystrophic Red Latosol | Red–Yellow Latosol | Nitisolic Red Latosol |
| pH(CaCl_2_) | 4.9 | 5.1 | 4.9 | 5.3 | 5.1 | 5.2 |
| H+Al (cmolc dm^3^) | 6.2 | 5.9 | 6.0 | 3.4 | 5.8 | 3.9 |
| K (cmolc dm^3^) | 0.6 | 2.0 | 0.6 | 0.4 | 0.6 | 0.3 |
| Ca (cmolc dm^3^) | 5.9 | 5.7 | 5.8 | 3.7 | 5.7 | 2.2 |
| Mg (cmolc dm^3^) | 2.4 | 2.0 | 2.2 | 1.5 | 2.0 | 0.6 |
| Al (cmolc dm^3^) | 0.1 | 0.0 | 0.0 | 0.0 | 0.01 | 0 |
| P (cmolc dm^3^) | 16.1 | 11.7 | 16.8 | 13.8 | 11.7 | 12.1 |
| Organic Matter (%) | 4.0 | 5.7 | 5.8 | 2.2 | 4.7 | 2.9 |

^1/^ Physical-chemical analyses were performed using soil layer samples from 0 to 20 cm.

^2/^ Köppen climate classification = Cfa, Humid subtropical climate; Cfb: Temperate oceanic climate; Am: tropical monsoon climate; and Aw: tropical savanna.


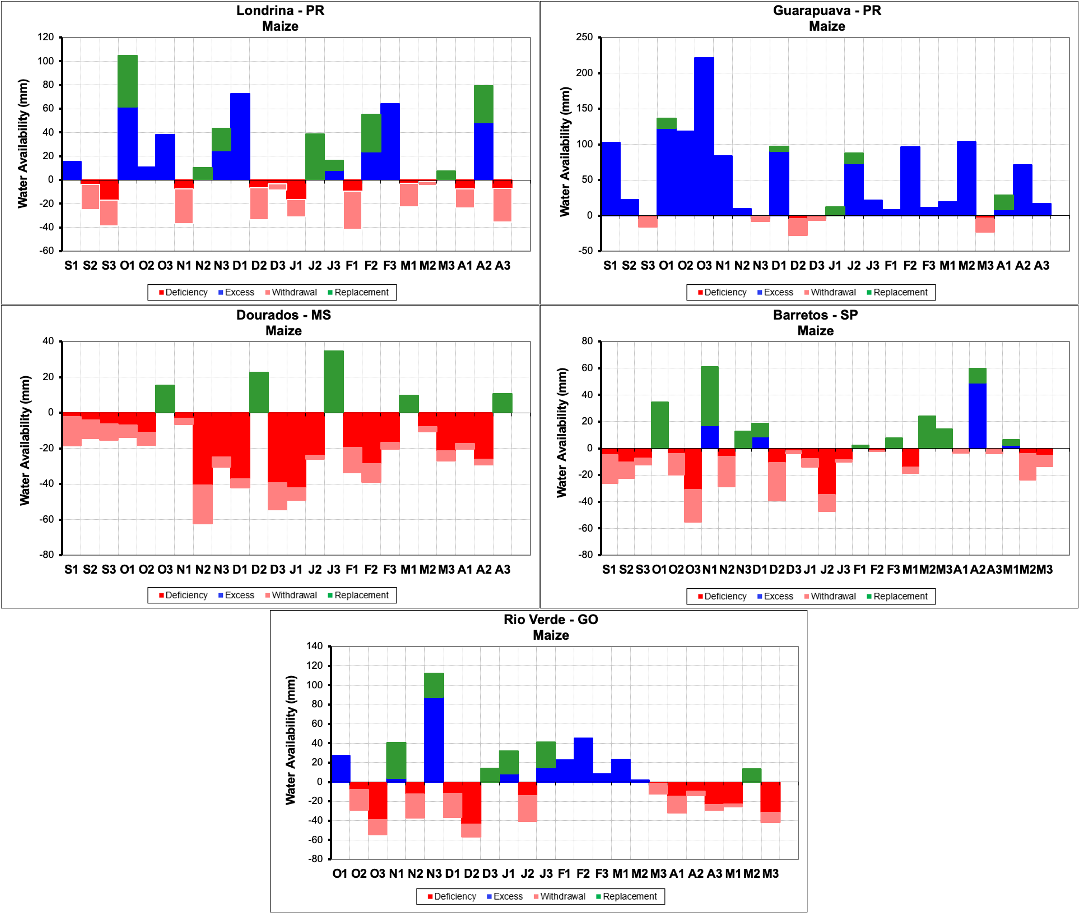


**Fig. S1.** Ten-day climatic water balance during the maize growing season at five Brazilian sites (Londrina, PR; Guarapuava, PR; Dourados, MS; Barretos, SP; and Rio Verde, GO). Bars show water availability (mm) partitioned into water deficit (red), soil-water withdrawal (light red), water surplus (blue), and soil-water replacement/recharge (green). The x-axis denotes ten-day periods within months from September (S) to April (A), where 1–3 indicate early, mid-, and late month, respectively.

**
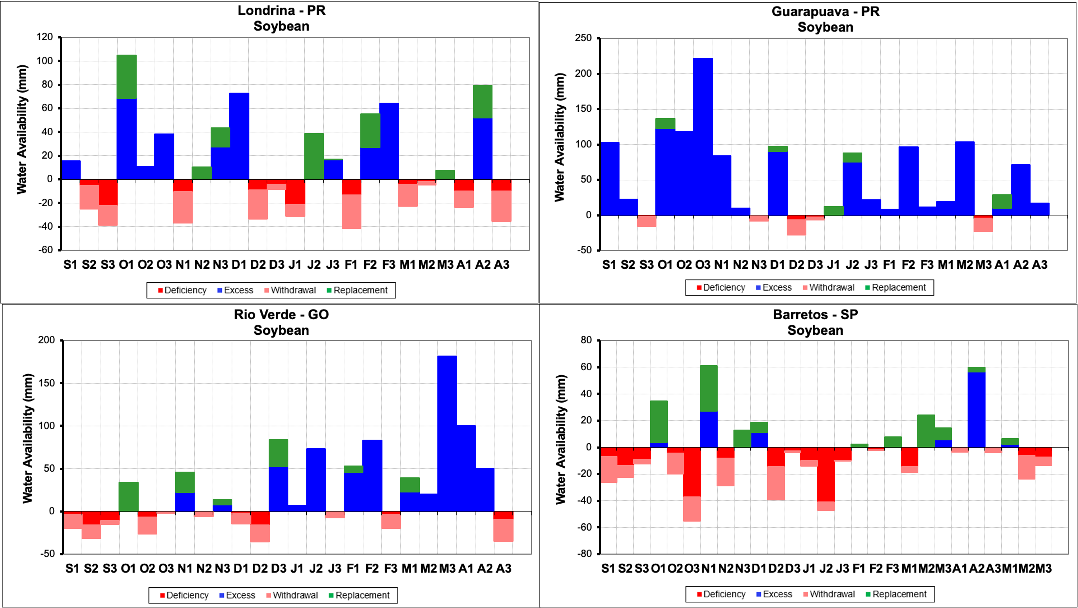
**

**Fig. S2**. Ten-day climatic water balance during the soybean growing season at four Brazilian sites (Londrina, PR; Guarapuava, PR; Rio Verde, GO; and Barretos, SP). Bars show water availability (mm) partitioned into water deficit (red), soil-water withdrawal (light red), water surplus (blue), and soil-water replacement/recharge (green). The x-axis denotes ten-day periods within months from September (S) to April (A), where 1–3 indicate early, mid-, and late month, respectively.


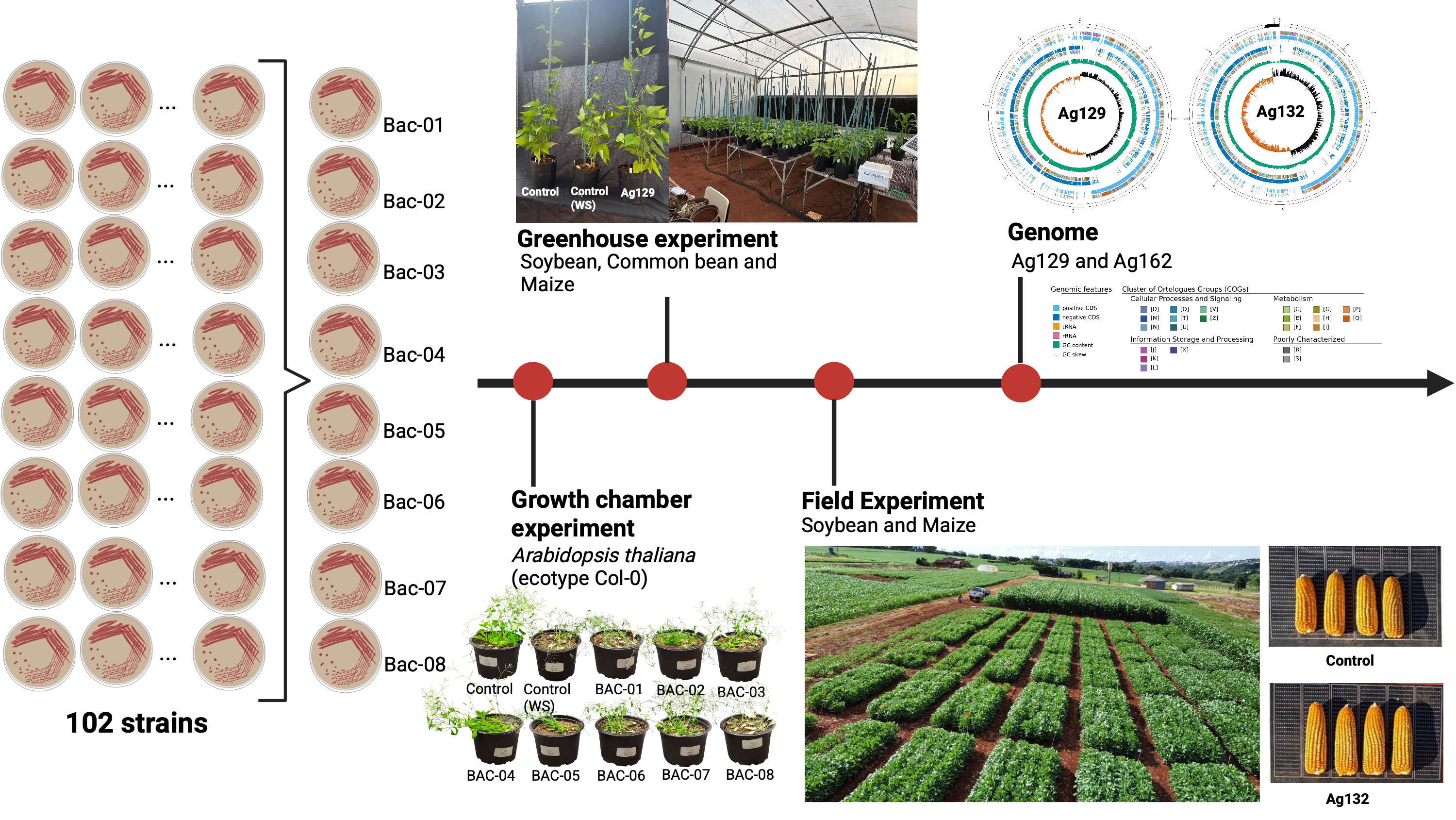


**Fig S3**. Workflow for the selection, phenotypic validation, and genomic characterization of Bacillus sp. strains that mitigate water deficit and promote plant growth.
